# Child feeding and human rights

**DOI:** 10.1186/1746-4358-1-27

**Published:** 2006-12-18

**Authors:** George Kent

**Affiliations:** 1 Department of Political Science, University of Hawai'i, Honolulu, Hawai'i 96822, USA

## Abstract

**Background:**

The human right to adequate food needs to be interpreted for the special case of young children because they are vulnerable, others make the choices for them, and their diets are not diverse. There are many public policy issues relating to child feeding.

**Discussion:**

The core of the debate lies in differences in views on the merits of infant formula. In contexts in which there is strong evidence and a clear consensus that the use of formula would be seriously dangerous, it might be sensible to adopt rules limiting its use. However, until there is broad consensus on this point, the best universal rule would be to rely on informed choice by mothers, with their having a clearly recognized right to objective and consistent information on the risks of using different feeding methods in their particular local circumstances.

**Summary:**

The obligation of the state to assure that mothers are well informed should be viewed as part of its broader obligation to establish social conditions that facilitate sound child feeding practices. This means that mothers should not be compelled to feed in particular ways by the state, but rather the state should assure that mothers are supported and enabled to make good feeding choices.

Thus, children should be viewed as having the right to be breastfed, not in the sense that the mother is obligated to breastfeed the child, but in the sense that no one may interfere with the mother's right to breastfeed the child. Breastfeeding should be viewed as the right of the mother and child together.

## Background

### Child feeding is political

In feeding young children, the primary parties are the mother and the child. But there are others with some interest and some influence in the situation. There is the father, and siblings. There is the extended family. There are friends. There is the local community. There are also doctors and nurses and other health professionals. Employers are affected. The local government may be concerned in some way, and possibly the national government, and even some international organizations. And there are also a variety of commercial interests.

Each of these parties has some interest in the child feeding relationship. All of them may feel or claim that they have a common interest in the health and well- being of the child, but they have other interests as well. The mother is, and indeed should be, concerned with her own health and comfort. Siblings may be jealous because of the attention paid to the newcomer. Some fathers may feel jealous as well. Both father and mother may be concerned about the mother's being drawn away from work in the field or the factory, or from caring for other family members. Older female relatives may try to influence the feeding process. Employers may be concerned with how breastfeeding takes the mother away from work, whether for minutes, days, or months. They may be concerned that publicly visible breastfeeding will distract other workers.

Health care workers may be concerned with the well-being of the child and the mother, but they also have other concerns. They may have only limited time and other resources for preparing and for assisting and enabling the new mother to breastfeed. Commercial interests may want to sell products, either to support breastfeeding (such as breast pumps or special clothing) or for alternatives to breastfeeding (such as infant formula, sterilization equipment). Government officials may be swayed in different directions, depending on which of these parties has the greatest influence on them.

These parties can influence one another's decisions in many different ways, through education, persuasion, money, affection. The child may not appear to be influential, but its birth and its behavior affect the mother's hormones, and provide a positive stimulus for breastfeeding. The hormones of pregnancy also cause proliferation of the ducts and alveoli of the mother's breasts, in preparation for production of colostrum and mature milk. As a result of the delivery of the placenta, the drop in progesterone starts the production of breast milk soon after the birth. Thus, lactation is the natural and direct result of pregnancy and birth.

Beyond that, the interests of the child may have an impact if he or she is represented by surrogates, others who have some capacity in the situation and who choose to speak and act in the child's behalf. Nevertheless, the child has little direct power in the relationship. It is particularly because of this extreme asymmetry in the power relationships that it is important to articulate the rights of the child.

### The human right to adequate food

The human rights of children with regard to their nutrition must be located within the broader context of the human right to adequate food in modern international human rights law and principles. The foundation lies in the *Universal Declaration of Human Rights*, which asserts, in article 25(1), that "everyone has the right to a standard of living adequate for the health and well-being of himself and his family, including food..." [1].

The right was reaffirmed in two major binding international agreements. In the *International Covenant on Economic, Social and Cultural Rights*, which came into force in 1976, article 11 says that "The States Parties to the present Covenant recognize the right of everyone to an adequate standard of living for himself and his family, including adequate food, clothing, and housing..." and also recognizes "the fundamental right of everyone to be free from hunger..." [2].

In the *Convention on the Rights of the Child*, which came into force in 1990, article 24 says that "States Parties recognize the right of the child to the enjoyment of the highest attainable standard of health..." (paragraph 1) and shall take appropriate measures "to combat disease and malnutrition...through the provision of adequate nutritious foods, clean drinking water, and health care" [3] (paragraph 2c).

The human right to adequate food is well established in international law. Even if the right had not been stated directly, it would be strongly implied in other provisions such as those asserting the right to life and health, or the *Convention on the Rights of the Child*'s requirement (in article 24, paragraph 2a) that States Parties shall "take appropriate measures to diminish infant and child mortality."

The UN's Committee on Economic, Social and Cultural Rights has issued *General Comment 12 (Twentieth session, 1999): The Right to Adequate Food (Art. 11) *(General Comment 12 1999), interpreting the meaning of the human right to adequate food [4]. It constitutes an authoritative contribution to international jurisprudence.

Several non-binding international declarations and resolutions have helped to shape the emerging international consensus on the meaning of the human right to adequate food as it applies to children. In October 1979 a joint WHO/UNICEF meeting on infant and young child feeding adopted a statement saying:

Breastfeeding is an integral part of the reproductive process, the natural and ideal way of feeding the infant and a unique biological and emotional basis for child development. This, together with its other important effects, on the prevention of infections, on the health and well-being of the mother, on child-spacing, on family welfare, on family and national economics, and on food production, makes it a key aspect of self-reliance, primary health care and current development approaches. It is therefore a responsibility of society to promote breastfeeding and to protect pregnant and lactating mothers from any influences that could disrupt it [5].

This was followed by several other international statements and agreements, including for example, the World Health Organization's *International Code of Marketing of Breast Milk Substitutes*, adopted in 1981, and the subsequent clarifying resolutions [6]. The code and the subsequent related resolutions may be accessed at the website of the International Baby Food Action Network [7]. The *Innocenti Declaration on the Protection, Promotion and Support of Breastfeeding *was agreed upon in 1990 [8] and reaffirmed in 2005 [9]. The International Labour Organization's *Maternity Protection Convention 103 *was revised in 2000, and became ILO Convention 183 [10]. The *Global Strategy for Infant and Young Child Feeding *was adopted by the World Health Organization in 2002 and published as a booklet in 2003 [11].

International human rights law has little to say explicitly about child feeding. However, article 24, paragraph (e) of the *Convention on the Rights of the Child *says that States Parties shall take appropriate measures...

To ensure that all segments of society, in particular parents and children, are informed, have access to education and are supported in the use of basic knowledge of child health and nutrition, the advantages of breastfeeding, hygiene and environmental sanitation and the prevention of accidents [3].

Also, article 24 says that States Parties shall "take appropriate measures to diminish infant and child mortality" [3].

## Discussion

### Right to food principles for children

The human rights approach can be helpful in analyzing and perhaps resolving policy issues relating to child feeding, but the human rights still need to be interpreted. A number of interested individuals, dissatisfied with prior attempts to formulate principles, agreed to discuss the issues through the Internet, beginning in May 1999 and continuing through to January 2000. The group's goal was to articulate a list of agreed principles relating to human rights and infant nutrition. After long hard discussion, the group formulated the Consensus Statement in Table 1.

**Table 1:** Consensus statement regarding the nutrition rights of infants

1. Infants have a right to be free from hunger, and to enjoy the highest attainable standard of health.
2. Infants have a right to adequate food, health services, and care.
3. The state and others are obligated to respect, protect, and facilitate the nurturing relationship between mother and child.
4. Women have the right to social, economic, health, and other conditions that are favorable for them to breastfeed or to deliver breast milk to their infants in other ways. This means that women have the right to:
a. Good prenatal care.
b. Basic information on child health and nutrition and the advantages of breastfeeding, and on principles of good breastfeeding and alternative ways of providing breast milk.
c. Protection from misinformation on infant feeding.
d. Family and community support in the practice of breastfeeding.
e. Maternity protection legislation that enables women to combine income-generating work with nurturing their infants.
f. Baby-friendly health facilities.
5. Women and infants have a right to protection from factors that can hinder or constrain breastfeeding, in accordance with:
a. The *Convention on the Rights of the Child*,
b. The *International Code of Marketing of Breast Milk substitutes *and related World Health Assembly resolutions,
c. The International Labour Organization's *Maternity Protection Convention Number 103 *and its subsequent revisions, and
d. The *Innocenti Declaration on the Protection, Promotion and Support of Breastfeeding*.
6. States, represented by their governments, have an obligation to:
a. Protect, maintain, and promote breastfeeding through public
b. educational activities,
c. Facilitate the conditions of breastfeeding, and
d. Otherwise assure that infants have safe access to breast milk.
7. No woman should be prevented from breastfeeding.

The process and outcome of this "Consultation on Human Rights and Infant Nutrition" were described in a report to the meeting of the United Nations System Standing Committee on Nutrition in Washington, D.C. in April 2000 [12,13].

The review of issues below makes it clear that there is a need for further discussion of the norms that should be established in relation to child feeding. Moreover, it should be understood that there is more to any human rights system than the articulation of norms. Rights imply concrete entitlements that need to be specified. Asserting that something is a right means not only that it is desirable, but also that people have enforceable claims on it. In addition to the rights holders and their rights, there must be well specified duty bearers who carry the obligations to the rights holders. And there must be systems of accountability to assure that the duty bearers do what they are supposed to do. The rights holders must have some means of recourse available to them, some effective institutional arrangements through which they can complain and try to have violations of their rights corrected [14].

### A sampling of issues

The feeding of children generally goes smoothly, particularly with the advice of appropriately trained health workers. However, there are times when views about appropriate methods of child feeding vary sharply. The difficulties sometimes are so serious and so extensive that they must be viewed as problems of society. The major issues, listed here, are all political in some way, and all can raise serious concerns about human rights.

#### (1) Coercion

Are there conditions under which the state may reasonably force a mother either to breastfeed or not breastfeed? The issue comes up, for example, when there is fear that the child might suffer from contaminants or infectious agents in the breast milk. Similarly, some women may be pressured to breastfeed because of fears that illness or death might result from the use of breast milk substitutes.

The view taken here is that under normal conditions the state should not interfere in the nurturing relationship between mother and child. The mother, in consultation with other family members, should be the one who decides how the child is to be fed. The mother has a range of choices, and is not to be limited to what some governmental agencies decide is the optimal diet.

However, the state may sometimes be justified in intervening in that relationship in extreme situations. These are situations in which there is clear evidence that the food (or other treatment) the mother intends to provide is highly likely to lead to extremely bad health outcomes for the child. If a mother wanted to treat her child's stomach-ache with a harmful dose of cyanide, we would want the state to block her. In all such cases where it is claimed that the situation is so extreme as to warrant state intervention, that would have to be based on clear and strong evidence of the danger.

In some circumstances the use of infant formula leads to much higher morbidity and mortality rates than are obtained with breastfeeding. In those situations we might accept a national government's prohibiting the use of infant formula, or controlling its use by, say, requiring a physician's prescription. However, in developed countries there is no clear and strong consensus that infant formula is so dangerous that women should not be allowed to use it to feed their infants. In this situation the appropriate action on the part of government might be to support educational campaigns and to assure that mothers make their decisions on the basis of objective and consistent information.

So long as there is great diversity in the community regarding the acceptability of infant formula, the tools of human rights cannot be used to force an agreement. It is only *in extremis *that the judgments of governments should override those of mothers, and then only when there is solid scientific evidence to support that judgment.

#### (2) Food safety

Many studies have shown that feeding infants with infant formula consistently results in worse health outcomes for infants than breastfeeding. There are risks of contamination of formula resulting from the fact that it is not a sterile product, risks of manufacturing errors, and risks associated with inappropriate preparation of the formula in the household. Even when it is produced and prepared properly, infant formula leads to inferior health outcomes because it lacks some of the key elements in breast milk, especially the factors that strengthen the infant's immune system [15].

The differences between breast milk and infant formula cannot be captured simply by comparing lists of ingredients. The March of Dimes summarizes the differences between breast milk and formula as follows:

Breast milk includes antibodies and other immune system substances that help protect a baby from illness. It contains growth factors, hormones and other substances that help a baby grow and develop at an appropriate rate. Breast milk also contains fatty acids that appear to promote brain development and, possibly, increase intelligence. Some formula makers add two of these fatty acids (DHA [docosahexaenoic acid] and ARA [arachidonic acid]) to their products. However, according to the AAP [American Academy of Pediatrics], the long-term benefits of formula enhanced with these fatty acids are not known [16].

Simply adding DHA and ARA into the mix does not guarantee they will work the way they do in breast milk.

The complexity of breast milk is illustrated by the fact that iron in breast milk is readily available (bioavailable) to the infant, but it is not readily available in infant formula. Some manufacturers have included much higher levels of iron in formula than is found in breast milk. The result can be toxic in various ways [17]. It should be recognized that breast milk is a complex, changing, living fluid, and not simply a collection of inert ingredients.

Sometimes the deficiencies of formula result in serious harm to infants and sometimes the harm is relatively small. How should one decide whether infant formula or any other breast milk substitute is only slightly unsafe to use, and thus a reasonable second-best choice, or so unsafe as to warrant government control? What should be done when there is no consensus on whether breast milk substitutes are sufficiently safe to use?

The risks associated with using breast milk substitutes could be compared with the risks of doing other kinds of things that we accept as normal, such as the risk of riding in cars. Some people might feel that children should not be exposed to any sort of risk under any conditions, but most people understand that all activities entail some amount of risk. One doesn't want to keep children in bed under guard all day long. The task is to find reasonable ways to balance different sorts of risk and different sorts of interests.

It has been estimated that in the United States about 720 infant deaths would be averted each year if all children were breastfed [18]. Does this mean that breast milk substitutes should be avoided? Apparently there is no consensus on this. Where some people are likely to judge the risks as high, and others as low, perhaps it is sensible to leave decisions to people's own judgments. However, people should be fully informed about the risks.

In some cases, extreme risks can be demonstrated on the basis of clear scientific evidence, and there are well established standards for judging what level of risk is acceptable risk. For example, it has been shown that in some developing countries the mortality rates for children who are fed with breast milk substitutes are far higher than they are for breastfed children [19]. Where the use of breast milk substitutes would be particularly dangerous, national legislatures could require that breast milk substitutes may be obtained only with a prescription from a physician.

Official standards for assessing the safety of breast milk substitutes are inadequate at both the global level (Codex Alimentarius) and the national level. To illustrate, most infant formulas are based on either cow's milk or soy "milk", and in the U.S. both of these ingredients are categorized as GRAS, which means Generally Regarded as Safe. Characterizing a product as GRAS means that the products do not need to be tested. Under this standard, basic infant formula that includes the required ingredients is simply assumed to be safe. The rule does not require any systematic assessment of whether the food is adequate, or whether it is as good as breastfeeding for the intended consumers.

The GRAS concept makes some sense when assessing whether a food item is reasonably safe to include in a diverse diet. It is wholly inadequate when that food item *is *the diet. Apart from infants, a single-item diet is rare in human experience. With a diverse diet, there is a good likelihood that errors or deficiencies in one part will be compensated or overcome by other parts of the diet. However, with a single-item diet there is no backup. Extreme care must be taken to assure that that item is of the highest possible quality.

There should be special concern not only because infant formula constitutes practically the entire diet, but also because it is for highly vulnerable infants. Showing that something has been safe for adults does not tell us whether it will be safe for infants.

Despite the fact that many questions have been raised about it, current standards do not require studies of the safety of soy-based formula for children. Even more concerns have been raised about the use of genetically modified soy in infant formula. Genetically modified soy has been categorized as GRAS, even when it is used as the basic component of children's entire diet.

The human rights approach tells us that governments should provide people with the information they need to make informed choices. In cases of extreme risk, where governments limit the options that are available, government is obligated to provide clear evidence on the nature of the risk. In all cases, it should be understood that people should have safe food, and beyond that, they have a human right to safe food. This means that people who feel that their food is not adequately safeguarded should have reasonable means for complaining and having the situation corrected.

#### (3) Adequacy and the "highest attainable standard of health"

Adequacy is an important concept in discussion of the human right to adequate food. The UN Committee on Economic, Social and Cultural Rights' *General Comment 12*, on the right to food, discusses the adequacy issue, but does not define it explicitly [4].

Both the *Universal Declaration of Human Rights *[1] and the *International Covenant on Economic, Social and Cultural Rights *[2] speak of the right to an "adequate" standard of living. However, the covenant also speaks of "the right of everyone to the enjoyment of the highest attainable standard of physical and mental health." What do these terms "adequate" and "highest attainable standard" mean?

The UN's Committee on Economic, Social and Cultural Rights has prepared a *General Comment on the right to health *[20]. Its paragraph 9 suggests that in current human rights law the right to "the highest attainable standard of health" depends in part on the level of resources available to the state. Governments of countries with more abundant resources should commit themselves to higher standards with regard to their people's health.

However, in relation to the right to an adequate livelihood, "adequacy" appears to mean that people should be assured of at least some minimum quality of life everywhere, even in very poor countries. All people everywhere should get what they need in order to live in dignity. Safety nets must be established to prevent health conditions from falling below a certain level, no matter how poor the country may be. "Adequacy" does not depend on the level of state resources.

On the basis of the landmark *Declaration of Alma-Ata *issued by the International Conference on Primary Health Care held in September 1978, one might say that the basic safety net that is required is that which would be established by a basic system of primary health care [21]. However, the differences between *adequate *and *highest attainable *still need to be clarified and reconciled.

Some would argue that the obligation to seek the highest attainable standard of health implies that breast milk substitutes should not be used except in very special circumstances, such as cases in which children have a rare metabolic disorder such as galactosemia. Others would argue that women should be free to use breast milk substitutes so long as they can be used in ways that are acceptable, feasible, affordable, sustainable and safe.

Can breast milk substitutes be regarded as adequate food for children? There is a clear consensus that some liquids such as tea or soda are not acceptable substitutes under any conditions. There is also a strong consensus that delivering breast milk through means such as wet nursing or the use of milk banks, or expressing and pasteurizing the mother's milk, are acceptable when conventional breastfeeding by the mother is not possible [11].

The major breast milk substitute of interest is infant formula. Many view it as adequate when it can be used under good conditions of sanitation etc., but inadequate under poor conditions. Some say all infant formula is always inadequate because it lacks the immune factors that breast milk provides to protect children from a broad range of diseases [15]. There is no clear global consensus on the adequacy of infant formula, either among mothers or among pediatricians and policymakers.

The question of what is adequate should be compared with the question of whether breast milk substitutes allow children to achieve "the highest attainable standard of health." If these standards are in fact different, which of them should be applied in relation to child feeding?

#### (4) Breastfeeding in public view

In some countries questions are raised about whether women have the right to breastfeed in public view. In the United States, many states have adopted laws asserting the right to breastfeed. Typically, the states that have adopted such laws assert that a mother is allowed to breastfeed her child in any location, public or private, where she is otherwise allowed to be.

The right to breastfeed in public is not meant to be reserved to government-owned facilities such as city parks or state court buildings. "Public" here is understood to include restaurants, stores, shopping malls, and sports stadiums, and other places frequented by the general public, even if they are privately owned.

In some places women have been harassed for breastfeeding children who were three or more years old. There is no evidence that breastfeeding older children does them any harm, so the basis for the objections is not clear. This issue is closely related to the issue of breastfeeding in public since objections usually seem to be raised only when the breastfeeding of older children takes place in the view of others. Hardly anyone complains about extended breastfeeding in private.

A detailed review of these and related issues may be found at the website of La Leche League [22].

#### (5) Maternity protection

*Maternity protection *is concerned with assuring that mothers who work outside the home, whether salaried or self-employed, have accommodations for feeding their children. This may come in the form of paid maternity leave, and also accommodations in the workplace, in the form of modified work schedules and appropriate spaces for child feeding. As indicated above, the International Labour Organization's *Maternity Protection Convention 103 *was revised in 2000 to become ILO Convention 183, known as the *Maternity Protection Convention, 2000*.

Women's right to breastfeed at their workplace has frequently been challenged. In a case in California, for example, a schoolteacher wanted to breastfeed her infant during her free hour. The Circuit Court held that the woman had a constitutional right to breastfeed, but that the state could abridge that right if there was a compelling state interest in doing so. The state was allowed to prevent her from breastfeeding because it had an interest in running efficient schools [23].

The World Alliance for Breastfeeding Action provides an overview of the issue of Breastfeeding Women and Work and a report on the status of maternity protection by country at its website [24].

Women in many different kinds of special circumstances need to be accommodated. For example, the US Air Force refused to provide a woman helicopter pilot with arrangements that would allow her to breastfeed her infant. With rare exceptions, the needs of children of women in prison have received practically no attention [25].

#### (6) Breastfeeding by women diagnosed as HIV-positive

There is an ongoing debate about how children of women who have been diagnosed as HIV-positive should be fed. Those who focus on the risks of transmission of the virus through breastfeeding generally advocate infant formula feeding, at least where that can be done in ways that are affordable, acceptable, feasible, sustainable and safe. However, many people say the focus should not be on the risk of transmission, but on the likely health outcomes. Some believe that exclusive breastfeeding would be the best choice, and some argue that the researchers have not done the studies that would be required to make informed choices. Some focus on the point that HIV-infected women, like uninfected women, have the right to the information they would need to make properly informed choices [26]. A nongovernmental organization called AnotherLook centers its work on the question of how children of mothers diagnosed as HIV-positive should be fed [27].

#### (7) Baby-friendly hospitals

In 1991 UNICEF and the World Health Organization launched the Baby-Friendly Hospital Initiative (BFHI) in an effort to ensure that all maternity facilities in which children are born (not only hospitals) become centers of breastfeeding support. A maternity facility can be designated as baby-friendly if it supports ten specific steps to support successful breastfeeding, and does not accept free or low-cost breast milk substitutes, feeding bottles or teats [28].

The UNICEF BFHI website provides data on the numbers of baby friendly facilities in different countries as of 2002. In many countries only a small portion of the maternity facilities qualify for baby-friendly status. As of September 2006 there were only 55 baby-friendly hospitals in the U.S [29]. There is a website that tracks BFHI progress in the United Kingdom [30].

#### (8) Promotion of breast milk substitutes

In the 1970s there was widespread alarm about the way in which the use of infant formula led to illness and death for children all over the world. The political campaigns against this led to the adoption of the *International Code of Marketing of Breast Milk Substitutes *by the World Health Assembly in 1981, and to a series of subsequent resolutions to further clarify and strengthen the code. Its purpose is not to prohibit the marketing of breast milk substitutes, but to prohibit their promotion, and to assure that women receive objective and consistent information about their advantages and disadvantages. Violations of the code are regularly documented by the International Baby Food Action Network (IBFAN) [7].

The Baby-Friendly Hospital Initiative, described above, has adherence to the code as one of its major requirements.

IBFAN has focused on challenging the promotion of breast milk substitutes by corporations. However, governments themselves sometimes violate the principles of the code. Governments commit violations not only by failing to force corporations under their jurisdiction to comply with the code, but also through their own distribution programs. For example, several countries distribute free infant formula through their social service programs. More than half the infant formula used in the US is distributed free through the federal government's Special Supplemental Nutrition Program for Women, Infants, and Children, commonly known as WIC [31].

In some cases, infant formula is provided as part of emergency relief supplies. However, many experts agree that it is generally wiser to assure that the mother is well fed and provided with appropriate support so that she can breastfeed successfully [32].

#### (9) Extra-jurisdictional obligations

Human rights work worldwide has focused on the ways in which the state, and thus the national government that represents it, has the primary obligation for assuring the realization of the human rights of people living under their jurisdiction. Human rights specialists are now giving increasing attention to extra-territorial obligations, or what would be described more precisely as extra-jurisdictional obligations [33]. States have obligations not only to their own people but also to all people everywhere.

With respect to children, in particular, this means, for example, that those who export breast milk substitutes and other infant foods have a measure of responsibility for their impact on the health of children in the receiving countries. Those who ship breast milk substitutes as part of their humanitarian assistance relief packages have responsibility for the consequences of its use. More generally, people's rights do not end at their national borders.

The rhetoric at international summit meetings frequently speaks of a global commitment to end hunger. However, as illustrated by the final report of the Millennium Task Force on Hunger in 2005, the international community is simply asked to make charitable donations [34]. The Millennium Task Force could have insisted that the international community has, or should have, a genuine legal obligation to end hunger. With over ten million children dying before their fifth birthday each year, year after year, the consequences are enormous. Children born into poor countries are not born into a poor world. Everyone, everywhere, has some measure of responsibility for all children everywhere [35].

#### (10) Women's rights to breastfeed vs. children's rights to be breastfed

There is widespread consensus regarding the right of women to breastfeed. However, there remains a knotty question: do – or should – children have the right to be breastfed? Some of the strongest advocates of breastfeeding argue that children should have this right, and thus – apart from special medical circumstances – women really should not have any choice in the matter.

Should mothers have no choice in the matter? What is the relationship between the mother's interest in breastfeeding and the child's interest in being breastfed? How do the mother's rights relate to the child's rights?

At times the mother and the child may have conflicting interests in relation to feeding. The conflict is raised in clear relief when it is argued that the child has a right not only to be well nourished but, more specifically, that the child has a right to be breastfed. Such a right could clash with the woman's right to choose how to feed her child.

Article 3 of the *Convention on the Rights of the Child *says, "In all actions concerning children...the best interests of the child shall be a primary consideration." Combining this with the observation that breastfeeding is better than alternative methods of feeding, some argue that children have a right to be breastfed [3].

In human rights law and principles, it is true that decisions must be based on consideration of the best interests of the child, but that is not the only consideration. Moreover, it is assumed that normally the parents judge what is in the child's best interests. The state should interfere in the parent-child relationship only in extraordinary situations, when there is compelling evidence that the parents are acting contrary to the best interests of the child.

Those who argue that the child should have the right to be breastfed center their argument on the point that breastfeeding is almost always best for the health of the child. While that may be true, it does not necessarily follow that breastfeeding must be mandated under human rights law. The task of human rights, and governance generally, is not to prescribe optimal behavior. Rather, their function is to establish outer limits, saying that people's behavior should not go beyond certain extremes. Thus, people are allowed to smoke and eat unhealthy food, even though it is not best for them.

By definition, human rights are universal; they do not vary from country to country, from place to place. However, national and local legislatures are free to formulate legal requirements appropriate to their particular local circumstances, provided they do not conflict with general human right rights law and principles.

The child has great interests at stake, but few resources to be used to press for preferred outcomes. Given the child's powerlessness and vulnerability, it is sensible to use the law to help assure that the best interests of the child are served. However, while it is surely appropriate to use the law to protect the child from outsiders with conflicting interests, the position proposed here is that it is not reasonable to use the law to compel an unwilling mother to breastfeed, or to prevent a willing mother from breastfeeding. For the purposes of framing appropriate law, the woman and child can be viewed as generally having a shared interest in the child's well-being. From the human rights perspective, the major concern is with protecting the woman-child unit from outside interference.

In many countries, the dominant view is that mothers should remain free to feed their children as they wish, in consultation with other family members. Outsiders are obligated to refrain from doing anything that might interfere with a mother's freely made and informed decision. Mothers should have objective and consistent information available to them so that they can make informed decisions. This is the approach taken in the *International Code of Marketing of Breast Milk Substitutes*. The code is not designed to prevent the marketing or use of formula, but to assure that parents can make a fully and fairly informed choice on how to feed their children. The main task is not to prescribe to women what they should do, but to remove all the obstacles to feeding their children in accordance with their own well informed choices.

Thus, the solution suggested here is that the mother and child together should be understood as having a type of group rights. *Breastfeeding is the right of the mother and the child together*. This could be expressed as the following principle:

• Children have the right to be breastfed, in the sense that no one may interfere with their mothers' right to breastfeed them.

This could replace principle 7, listed earlier: "No woman should be prevented from breastfeeding."

This proposed formulation means that the mother-child pair, taken together, have certain rights in relation to outside parties, such as rights to certain kinds of information and services, and the rights to be protected from undue influences from outside interests. It does not say that women are obligated to breastfeed their children. It does not invite the state to intervene in the relationships between mothers and their children.

The principles proposed here (with the revised number 7) do not give priority to the mother or to the child, but instead try to forge a sensible balance between their interests. The principles are based on the concept that mothers should not be legally obligated to breastfeed, but rather they should be supported in making their own informed choices as to how to feed their children.

#### National Legislation

While human rights law and principles are intended to be universal, they are deliberately stated in generalized form in order to leave room for interpretation at national and sub-national levels. This task of interpretation may be accomplished through the formulation and adoption of national law that is designed to support implementation of global human rights at the national level.

When states become parties to international human rights agreements, they are expected to elaborate their understandings of those obligations by spelling them out in their own national law. Indeed, there is a positive obligation to do this, described, for example, in article 2, paragraph 2 of the *International Covenant on Civil and Political Rights *[36], and also in *General Comment 12*, paragraph 29 [4].

Thus, children' rights with regard to their feeding, the correlative obligations of national government agencies and others, and the mechanisms of accountability should be spelled out in national legislation. In preparing rights-based legislation on child feeding, several major steps should be taken:

(1) Review existing national and sub-national (e.g., provincial) legislation relating to child feeding;

(2) Identify the major departments and agencies of government that have, or might be assigned, responsibility for issues relating to child feeding;

(3) Survey legislation on comparable issues that has been adopted in other jurisdictions;

(4) Identify what issues relating to child feeding are of interest in the particular country, such as the sampling of issues listed above;

(5) Formulate basic principles regarding child feeding. Although there is as yet no global consensus on the proposed principles described in earlier sections of this paper, these proposals can be used as the basis for formulating principles that can be agreed upon at the national level;

(6) Prepare a draft of new legislation for rights-based child feeding. Structure this legislation so that it clearly articulates: (a) the relevant rights of both children and their mothers (or other caretakers); (b) the corresponding obligations of government agencies and others; (c) the mechanisms of accountability;

(7) Refine this draft on the basis of broad consultations within the government, with concerned nongovernmental organizations, and with the general public;

(8) Campaign for passage of this proposed legislation through a broad program of public education and debate.

New framework legislation on child feeding may be embedded into more comprehensive legislation on the care of children. It may incorporate existing legislation relating to child feeding directly or by making references to it. This legislation should take a large view and establish the means for steady improvement in child feeding. It should set clear goals and provide means for monitoring progress toward the goals. For example, targets could be set out for increasing the proportion of mothers who exclusively breastfeed for at least six months, and for increasing the number of Baby Friendly Hospitals.

The human rights of children should be concretized into specific entitlements at the national level. For example, one might say that all children are entitled to baby-friendly maternity facilities, and thus all pregnant women are entitled to have access to a baby-friendly maternity facility within a half hour's travel from their homes. These conditions could be defined in terms of the *Ten Steps to Successful Breastfeeding *for Baby-Friendly Hospitals. The law also should provide for remedies for those who did not receive what they were entitled to, whether through administrative or judicial procedures. The law could establish specific remedies. For example, the law could require that any woman who did not have access to a baby-friendly maternity facility near her home could be entitled to the services of a lactation counselor at no cost.

Similarly, the right to breastfeed in public could be backed up by various devices. Managers of spaces open to the public such as shopping malls and concert halls could be provided with appropriate instructions to prevent harassment by their employees, and they could be instructed on how to handle complaints about women breastfeeding. Complaints about harassment could be invited by an appropriate government agency, an ombudsman's office, or a nongovernmental organization. In the state of Hawai'i, for example, the law says, "It is a discriminatory practice to deny, or attempt to deny, the full and equal enjoyment of the goods, services, facilities, privileges, advantages, and accommodations of a place of public accommodations to a woman because she is breastfeeding a child." The law provides that, "Any person who is injured by an unlawful discriminatory practice under this part may bring proceedings to enjoin the unlawful discriminatory practice, and if the decree is for the plaintiff, the plaintiff shall be awarded reasonable attorneys' fees, the cost of suit, and $100" [37].

The law could mandate that those who harass breastfeeding mothers must participate in an educational program, perhaps provided by a breastfeeding advocacy group under contract with the government. It is probably wiser to respond to this sort of issue with educational programs rather than with threats of punishment.

IBFAN has developed a model law for countries that want to adopt the principles of the *International Code of Marketing of Breast Milk Substitutes *into their national law [38]. Of course countries that draw guidance from this model also should consult the specific legislation that has been adopted in other countries, and they should make adaptations to suit their own local circumstances.

Similarly, there are collections of laws regarding maternity protection and breastfeeding in public that can be consulted to help in formulating aspects of national law relating to child feeding. The World Health Organization's International Digest of Health Legislation may be helpful as well [39].

Much can be learned about these issues from the United Kingdom's Baby Feeding Law Group, "Working to bring UK baby food laws into line with international standards," through its website [40]. Comparable advocacy groups could be created in other countries, focusing on the child feeding issues that are of particular concern to them.

Child feeding is a highly political issue, with many different parties pushing and pulling in different directions to pursue their own interests. Children's interests should be protected through clear rights stated in terms of specific entitlements, named agencies with specific obligations for assuring the realization of those rights, and effective mechanisms of accountability. Through this sort of well-crafted law, the nation can spell out its commitments to assuring that all of its children are well nourished.

#### Obligations of the state

As explained by the United Nations Committee on Economic, Social and Cultural Rights:

The right to adequate food, like any other human right, imposes three types or levels of obligations on States parties: the obligations to *respect*, to *protect *and to *fulfil*. In turn, the obligation to *fulfil *incorporates both an obligation to *facilitate *and an obligation to *provide*. The obligation to *respect *existing access to adequate food requires States parties not to take any measures that result in preventing such access. The obligation to *protect *requires measures by the State to ensure that enterprises or individuals do not deprive individuals of their access to adequate food. The obligation to *fulfil *(*facilitate) *means the State must pro-actively engage in activities intended to strengthen people's access to and utilization of resources and means to ensure their livelihood, including food security. Finally, whenever an individual or group is unable, for reasons beyond their control, to enjoy the right to adequate food by the means at their disposal, States have the obligation to *fulfil (provide) *that right directly [4] (para 15).

With regard to the feeding of infants and young children, this may be interpreted as follows:

The obligation to *respect *means that the state should not interfere in the nurturing relationship between mother and child. However, exceptions might be made to this rule in exceptional situations when scientifically justified and appropriate safeguards are taken against abusive interference.

The obligation to *protect *means that the state must protect the nurturing relationship from interference by others. The *International Code of Marketing of Breast Milk Substitutes *and the subsequest related resolutions of the World Health Assembly may be understood as a measure through which states can carry out their obligation to protect.

The obligation to fulfill in the sense of *facilitate *means that the state is obligated to do positive things to support the nurturing relationship between mother and child. This implies that the state must do things such as conduct research and provide information in appropriate forms so that mothers will have the objective and consistent information they need in order to made informed choices. It also means the state should work to eliminate obstacles to breastfeeding by assuring adequate maternity protection, and by supporting the creation of baby-friendly birthing facilities. States may also support wet nursing and the operation of milk banks in order to facilitate the provision of breast milk through means other than conventional breastfeeding by the mother.

The state's obligation to fufil the right in the sense of *provide *is limited because it is not equipped to breastfeed infants directly. The state is capable of providing breast milk substitutes. However, that should be limited to cases of special needs because it may have the effect of promoting breast milk substitutes, thus violating the principles of the *International Code of Marketing of Breast Milk Substitutes *and subsequent related resolutions of the World Health Assembly.

## Summary

There is widespread concern that mothers might make unwise choices with regard to feeding their children. We then have two basic options: either have society override the mother's choice, or find ways to support the mother so that she makes wise choices. In my view, the first approach is disempowering, while the second is empowering for women. If women are enabled by being given good information, having all the obstacles to breastfeeding eliminated, and having appropriate social support (such as maternity leave) they are likely to make good choices.

Rather than have the state make decisions for them, citizens in a democracy prefer assurances that nothing impedes them from making their own decisions. To the extent possible we should be free to choose, and that includes being free to some extent to make what others might regard as unwise or sub-optimal decisions.

Instead of focusing on the mother as if she were a more or less isolated decision-maker, some empasize the role of her surrounding society and culture. As James Akré puts it, it is not women but entire societies who decide how infants are to be fed. If feeding patterns are to be changed, that must be done through changes at the societal level [41]. Analysis at the societal level leads Elisabet Helsing and others to strong conclusions about the obligations of the society to facilitate breastfeeding as a consequence of its obligations to both the mother and the child [42]. As Michael Latham put it, "almost all mothers living under optimally baby-friendly conditions would make the choice to breastfeed" [43] (p. 416).

The core of the debate lies in differences in views on the merits of infant formula. Some people view formula as a good modern convenience while others view it as close to poison. Others are arrayed somewhere in between. In contexts in which there is strong evidence and a clear consensus that the use of infant formula would be seriously dangerous, it might be sensible to adopt rules limiting its use. However, until there is broad consensus on this point, the best universal rule would be to rely on informed choice by mothers, with their having a clearly recognized right to objective and consistent information on the risks of using different feeding methods in their particular local circumstances. The obligation of the state to assure that mothers are well informed should be viewed as part of its broader obligation to establish social conditions that facilitate sound child feeding practices.

## Competing interests

The author(s) declare that they have no competing interests.
